# Functional divergence of an immune receptor complex in *Nicotiana benthamiana*

**DOI:** 10.1093/plcell/koad186

**Published:** 2023-06-29

**Authors:** Bradley Laflamme

**Affiliations:** Assistant Features Editor, The Plant Cell, American Society of Plant Biologists, USA; Department of Molecular Genetics, University of Toronto, Toronto, ON, Canada

In recent years, plant research has been marked by an increasing appreciation for the receptor networks that underpin everything from development to disease resistance. In Arabidopsis (*A. thaliana*), transmembrane receptors in the leucine-rich repeat receptor kinase (LRR-RK) class form a tightly regulated network through physical interactions to coordinate a broad range of physiologically crucial functions (Smakowska-Luzan et al. 2018). The co-receptor BAK1 is central to this network, as it interacts with many other LRR-RKs to regulate a range of important processes, including immunity (Halter et al. 2014). The BAK1 interactome is well-established in Arabidopsis but comprehending how BAK1 interactions have diversified across the plant kingdom is our next step forward.

In this issue, **Liu and colleagues (Liu et al. 2023)** showcase a comparative analysis of LRR-RK orthologs from Arabidopsis and the model tobacco plant *Nicotiana benthamiana*. Using a virus-induced gene silencing approach in *N. benthamiana*, the group screened for LRR-RKs that regulate reactive oxygen species production in response to flg22, an immune-eliciting epitope of bacterial flagellin. Notably, silencing of 2 *N. benthamiana* orthologs of the Arabidopsis receptor BIR2 (NbBIR2 and NbBIR2L) had a negative impact on reactive oxygen species production, suggesting that these orthologs positively regulate immunity. Consistent with this hypothesis, Nb*bir2* mutants were more susceptible to bacterial and oomycete pathogens. These findings were particularly interesting because the Arabidopsis ortholog, AtBIR2, negatively regulates immunity through its interaction with AtBAK1 (Halter et al. 2014). These observations prompted Liu and colleagues to further explore the functional divergence of BIR2 in *N. benthamiana*.

Using immunoprecipitation followed by liquid chromatography-tandem mass spectrometry, the group identified NbBAK1 as a strong NbBIR2 interactor in both the presence and absence of flg22—a finding they validated with both co-immunoprecipitation and split-luciferase complementation. In contrast, the interaction between AtBIR2 and AtBAK1 declined with flg22 treatment using the same protocols, consistent with the role of AtBIR2 as a negative regulator of AtBAK1-activated immunity. The constitutive interaction between NbBIR2 and NbBAK1 also did not require BAK1 kinase activity, once again in contrast to AtBIR2, which does require BAK1 kinase activity to negatively regulate immunity (Halter et al. 2014).

NbBAK1 also appeared to be required for stabilizing NbBRI2, as a Nb*bak1* null mutant was unable to accumulate wild-type levels of the protein. Liu and colleagues thus investigated whether NbBAK1 shields NbBIR2 from degradation. Among the NbBIR2 interactors identified using liquid chromatography-tandem mass spectrometry were 2 ubiquitin ligases, NbSNIPER2a and NbSNIPER2b. Co-immunoprecipitation and co-expression analyses confirmed that NbSNIPER2a and NbSNIPER2b interact with and target NbBIR2 for degradation. However, simultaneous overexpression of NbBIR2 and NbSNIPER alongside NbBAK1 revealed that NbBIR2 accumulation is recovered when NbBAK1 is present. The authors close their study by showing that the NbBAK1 cytoplasmic domain can indeed outcompete NbSNIPERs for binding to the cytoplasmic domain of NbBIR2, thereby preventing its degradation. The group proposes a model whereby NbBIR2 can be readily degraded by ubiquitin ligases in the absence of an immune response. However, upon immune activation, NbBIR2 can be shielded from degradation by BAK1, allowing for this positive regulator of immunity to bolster plant defense (see Fig.).

**Figure.**
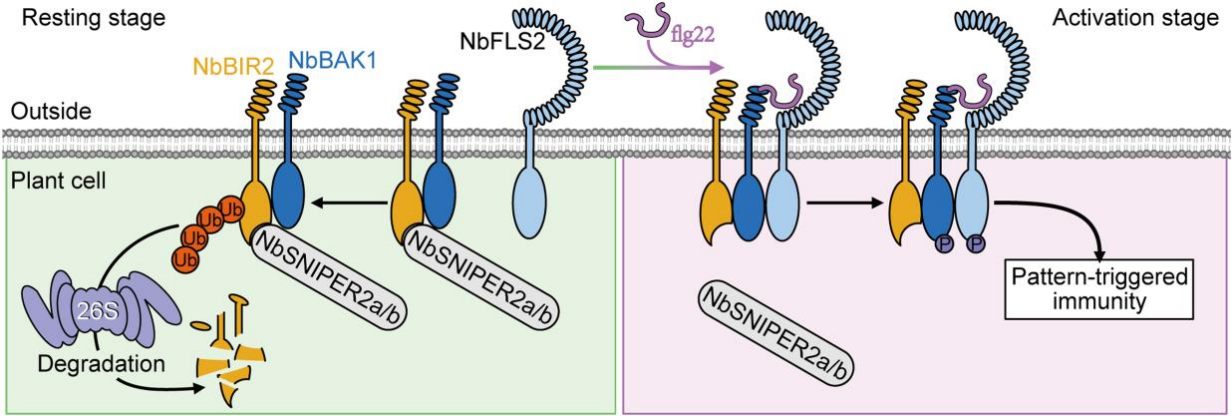
Proposed model for BIR2 activity in *N. benthamiana*. Before immune activation, NbBIR2 is more easily bound by NbSNIPER2a/b, leading to proteasomal degradation to avoid unwanted immune activation. Upon immune activation, NbBAK1 competes for NbBIR2 binding, enabling NbBIR2 to activate immunity (referred to as Pattern-Triggered Immunity in the figure). Reprinted from Liu *et al*. (2023) Figure 7.

With this study, Liu and colleagues highlight a powerful example of functional divergence between the LRR-RKs of 2 model plants. Like its Arabidopsis ortholog, NbBIR2 is an important LRR-RK that plays a role in immune regulation; but its relationship to BAK1 and its general effect on immunity are essentially inverted compared with AtBIR2. Cumulatively, these data suggest alternative—but no less effective—strategies for using receptors like BIR2 to properly respond to pathogens. More broadly, this study showcases the value of probing the LRR-RK network of *N. benthamiana* for valuable new insights into receptor function. Plant immunity aficionados will no doubt remember that the discovery of intracellular receptor networks governing immunity was made using *N. benthamiana* through investigating a special class of receptors not found in Arabidopsis (Wu et al. 2017). Who knows what else those tobacco plants are hiding?
